# Gastric Calcifying Fibrous Tumor: An Easy Misdiagnosis as Gastrointestinal Stromal Tumor—A Systemic Review

**DOI:** 10.3390/medicina56100541

**Published:** 2020-10-14

**Authors:** Meng-Ko Tsai, Hung-Yi Chen, Ming-Lung Chuang, Chun-Wen Chen, Gwo-Ping Jong

**Affiliations:** 1 Division of Rheumatology, Department of Internal Medicine, Taichung Armed Forces General Hospital, Taichung 41152, Taiwan; raymond42770@gmail.com; 2 Division of Rheumatology, Department of Internal Medicine, National Defense Medical Center, Taipei 11490, Taiwan; 3 Department of Pharmacy, China Medical University, Taichung 40402, Taiwan; hungyi@mail.cmu.edu.tw; 4 Department of Pharmacy, China Medical University Beigang Hospital, Yunlin County 65152, Taiwan; 5 Division of Pulmonary Medicine, Department of Internal Medicine, Chung Shan Medical University Hospital, Taichung 40201, Taiwan; cshy1292@csh.org.tw; 6 School of Medicine, Chung Shan Medical University, Taichung 40201, Taiwan; 7 Department of Radiology, Taichung Armed Forces General Hospital, Taichung 41168, Taiwan; lylmed066@gmail.com; 8 Department of Internal Medicine, Chung Shan Medical University Hospital, Taichung 40201, Taiwan; 9 Department of Internal Medicine, Chung Shan Medical University, Taichung 40201, Taiwan

**Keywords:** submucosal tumor, calcifying fibrous tumor, gastrointestinal stromal cell tumors

## Abstract

*Background and Objectives:* Calcifying fibrous tumor (CFT) in the stomach is extremely rare and is easily misdiagnosed as a gastrointestinal stromal tumor (GIST). This study aims to determine the best method to differentiate between gastric CFT and GIST after a systemic review and meta-analysis. *Materials and Methods:* A systematic search of articles using electronic databases (MEDLINE, EMBASE, and LILACS) was conducted and resulted in 162 articles with 272 CFT cases published from January 1988 to September 2019. *Results:* Of these cases, 272 patients, 60 patients with gastric CFT (32 men and 28 women, mean age 49.2 years) were analyzed. The mean tumor size was 2.4 cm in patients with gastric CFT. Both endoscopic ultrasound (EUS) and computed tomography (CT) findings revealed well-defined (100% vs. 77.8%), heterogeneous (100% vs. 77.8%), iso-hypoechoic (71.4% vs. 33.3%), and calcified (85.7% vs. 77.8%) lesions, respectively. The majority of patients (53.3%) were symptomatic, with the most common symptom being abdominal discomfort (55.6%). None of the patients with gastric CFT showed recurrence after treatment, and most patients received nonendoscopic treatment (56%, *n* = 28/50). Both age and tumor size were statistically significant in patients with gastric CFT than GIST (49.2 vs. 65.0 years and 2.4 vs. 6.0 cm; both *p* < 0.001). The ratio of children among patients with CFT (5%) and GIST (0.05%) was also significantly different (*p* = 0.037). The calcification rates of gastric CFT had significantly higher calcification rates than GIST on images of EUS and CT (85.7% vs. 3.6% and 77.8% vs. 3.6%; both *p* < 0.001). *Conclusions:* Compared with patients with GIST, patients with gastric CFT were younger, had smaller tumor size, and were symptomatic. Furthermore, gastric CFT was well-defined, heterogeneous in the third layer, and had high calcification rates on the images.

## 1. Introduction

A submucosal tumor (SMT) is a term used by endoscopists to depict any protuberance with intact mucosa. The etiology of most SMTs cannot easily be determined by esophagogastroduodenoscopy (EGD), computed tomography (CT), magnetic resonance image, or barium radiography [1]. However, experienced endoscopists may sometimes speculate on the etiology of SMT based on the size, shape, and overall appearance of the tumor. The incidence of gastric SMT is around 0.3% of routine EGD [1]. Typical SMTs are gastrointestinal stromal cell tumors (GISTs), leiomyomas, and schwannomas, and the most common one is GIST (80%) [2,3]. A calcifying fibrous tumor (CFT), another uncommon mesenchymal tumor, could occur in the stomach and be easily misdiagnosed with GIST [4]. CFT is a rare benign fibrous tumor. It is found as a solitary tumor or multiple lesions. The stomach seems to be the most common location of CFT [5]. The treatment of gastric CFT is simple; excision of the tumor is the choice of treatment with excellent prognosis [5]. However, gastric CFTs are under-recognized because gastroenterologists, surgeons, and pathologists are unfamiliar with them. Gastric CFTs could be treated with local incision or endoscopic submucosal dissection (ESD). Many cases have undergone open surgery, such as wedge resection or gastrotomy [5]. The characteristics of gastric CFT seem to be different from GIST. Therefore, an investigation of the gastric properties of CFTs might broaden our knowledge about the cause of SMT.

There are no original studies regarding the comparison of CFT and GIST until now. Gastric CFT has been only infrequently discussed and sparsely investigated in the literature. Whether it could be differentiated from GIST remains uncertain, especially in preoperative surveys. Therefore, this study evaluates the clinical characteristics, including age, tumor size, stomach layer, symptoms, treatment choice, and gastric CFT imaging findings from the literature search, and compared these data with GIST.

## 2. Materials and Methods

### 2.1. Patients

Our study followed the methods used in the Preferred Reporting Items for Systematic Reviews and Meta-Analyses. Eligibility criteria were specified clearly to ensure that studies were selected in a systematic and unbiased manner. We included case series studies and retrospective cohort studies. The patient population was defined as adults or children (≤18 years old) diagnosed with CFT in any part of the stomach. A search was conducted to identify studies that involved patients with CFT in the stomach. Additionally, studies had to include CFT found at autopsy.

### 2.2. Information Sources

We identified studies by searching electronic databases and then consulting with experts in the field. The search was performed using multiple databases, including PubMed (1988 to September 2019), EMBASE (1990 to September 2019), and LILACS (1987 to September 2019). A proficient research librarian provided the following predefined list of search terms and medical subject headings: calcifying fibrous tumor, calcifying fibrous pseudotumor, fibrous pseudotumor, childhood fibrous tumor with psammoma bodies, children fibrous tumor with psammoma bodies, pediatric fibrous tumor with psammoma bodies. No language restrictions were imposed on these searches.

### 2.3. Study Selection

A double review process was applied to review the abstracts of all citations verified in the searches. After duplicate outcomes were removed, potentially relevant articles were included based on their full text. Additional studies were obtained from references of the selected articles. Controversial results were resolved by consensus among the senior authors (J-GP). Only studies fulfilling the eligibility criteria were included in this systematic review.

### 2.4. Synthesis of Results

The intended summary measure of treatment or imaging findings might have differed from that used in some studies. Therefore, we prespecified the summary measure of treatment and imaging findings. We divided these treatments into four categories: intervention (endoscopic treatment), diagnostic treatment (including excision, local incision, and total biopsy), minor operation (resection), and major operation (Billroth I procedure, partial gastrectomy, and sleeve gastrectomy). For imaging findings with different interpretation results, all the image findings were re-evaluated by our radiologist. Additionally, calcification was divided into two groups in CT: macrocalcification (coarse areas of calcification that are greater than 1 mm in size) and microcalcification (punctate areas of calcification that are less than 1 mm in size).

### 2.5. Statistical Analysis

Continuous variables were expressed as valid percentages and mean values with the standard deviation. For univariate analyses, *t*-tests were performed. Differences were assessed by the chi-squared test or Fisher’s exact test. A *p*-value of <0.05 was considered statistically significant. SPSS 19.0 statistical software (SPSS, Chicago, IL, USA) was used to perform all statistical calculations.

## 3. Results

### 3.1. Study Selection Process

**Figure 1:**
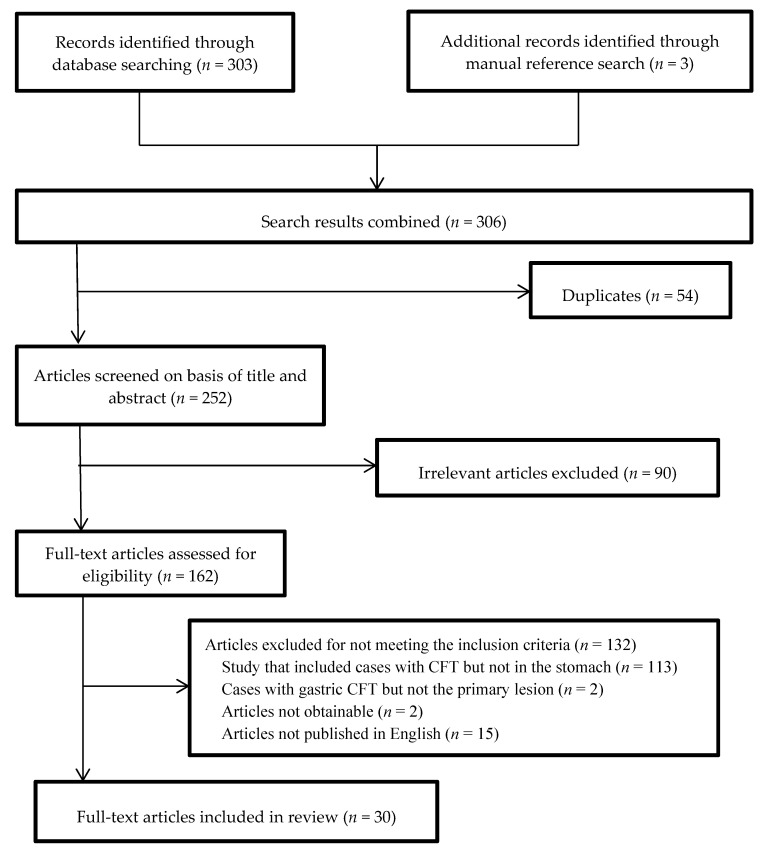
Flow diagram of study selection process.

A total of 306 records were identified (Figure 1). Three additional records were identified by manual reference searches. After removing duplicates (54 records), we screened 252 records based on the title and abstract. From these, 90 records with subjects that were different or irrelevant were eliminated. We assessed 162 full-text articles according to the inclusion. One hundred and thirty-one articles were excluded for the following reasons: a study that included cases with CFT but not in the stomach (*n* = 113), cases with gastric CFT but not the primary lesion (*n* = 2), articles not obtainable (*n* = 2), and articles not published in English (*n* = 15). No randomized controlled trial was included in the review. Finally, 30 full-text articles [5,6,7,8,9,10,11,12,13,14,15,16,17,18,19,20,21,22,23,24,25,26,27,28,29,30,31,32,33,34] were included (Table 1).

### 3.2. Clinical Characteristics of All Patients

Most eligible studies (85%) were published in the last decade. The designs of the studies we included were retrospective studies (*n* = 5) and case reports (*n* = 25). A total of 60 patients with gastric CFT were evaluated in this study, ranging from 5–77 years. In most cases, the follow-up was conducted for >six months.

### 3.3. Age, Children Ratio, and Size

**Figure 2:**
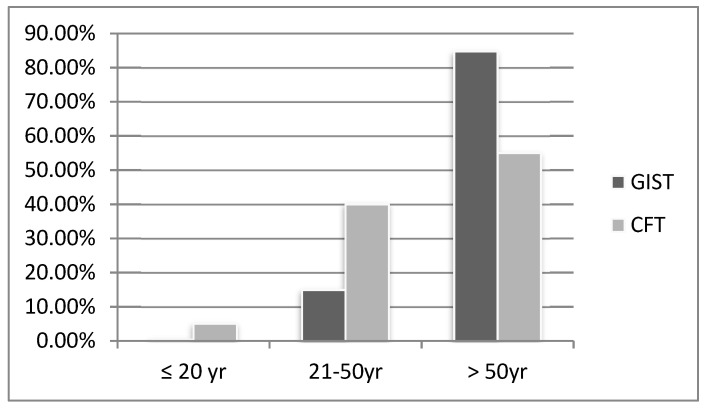
The age distribution between gastric calcifying fibrous tumors and gastrointestinal stromal cell tumors.

The mean age was 49.2 ± 13.9 years. The age distribution compared with GIST is shown in Figure 2. When we compared age with that of GIST (median age: 65 years) [35], it was statically significant (*p* < 0.001). Fifty-eight cases mentioned the size of the tumor with a mean tumor size of 2.4 cm, which, compared with GIST, was also statically significant (*p* < 0.001). Three cases (5%) were children, and the ratio was significantly different from GIST (0.5%) [35] (*p* = 0.037) (Table 2).

### 3.4. Symptoms

Seven different leading symptoms for gastric CFT were detected. Most patients were symptomatic (*n* = 32, 53.3%). Thirteen patients mentioned the period of symptoms, and three of them were acute, and 10 were chronic. Ten patients had ≥two symptoms, seven patients had double symptoms, and three patients had triple symptoms. Therefore, a total of 45 symptoms were analyzed. The most common symptom was abdominal discomfort (55.6%, *n* = 25/45). This symptom was followed by abdominal pain (17.8%), and abdominal distention (8.9%). The distribution of the leading symptoms is shown in Table 3.

### 3.5. Location and Layer of the Stomach

Thirty-one patients mentioned location, and most patients’ tumors (74.2%) were in the body of the stomach. Thirty-four patients mentioned the layer of the tumor in the stomach. There are five distinct layers of the stomach on endoscopic ultrasound (EUS). Most tumors were located in the submucosa (*n* = 19, 55.9%). Five patients’ tumors were in the mucosa/muscularis mucosa, eight patients’ tumors were in the muscularis propria, and two patients’ tumors were in the serosa (Table 2).

### 3.6. Endoscopic Ultrasound

Fourteen patients received EUS, and seven patients were excluded for imaging analysis because there was no picture to evaluate. The tumors of all patients (*n* = 7) were heterogeneous and well-defined. Most patients had tumors that were iso-hypoechoic (71.4%) and acoustic shadowing (71.4%) (Table 2).

### 3.7. Computed Tomography

Fourteen patients received CT, and five patients were excluded (two patients because there was no picture to evaluate, and three patients because the tumor could not be detected). The tumors of the remaining nine patients were mostly were well-defined (77.8%), heterogeneous (77.8%), homo-hyperdense (66.7%), and with calcification (77.8%). In patients with calcification, most (85.7%) were macrocalcification (coarse calcification) (Table 2).

### 3.8. Calcification

Sixteen patients received EUS or abdominal CT, and two patients received both CT and EUS. Therefore, 14 patients received EUS or CT, and 12 (85.7%) had calcification. The overall calcification rate was 85.7% (Table 2). We compared the calcification rate with GIST by the chi-square test and found that they were significantly different (*p* < 0.001) (Table 4).

### 3.9. Choice of Treatment

A total of 50 patients mentioned the choice of treatment (10 patients were excluded because six did not mention the choice of treatment, one was diagnosed at autopsy, and three were incidental diagnoses of CFT and were excluded from the treatment analysis). Most patients received a minor operation (*n* = 23, 46%), followed by intervention (36%), a major operation (12%), and diagnostic treatment (8%). More than half the patients (*n* = 32, 64%) received nonendoscopic treatment.

## 4. Discussion

Our study is the first to discuss gastric CFT as a differential diagnosis of GIST. We systematically reviewed the size, symptoms, imaging findings, and treatments of gastric CFT. We conducted a systemic search of the articles and reviewed 30 publications. We found that (i) the most common findings in EUS were heterogeneous (100%, *n* = 7/7), iso-hypoechoic (71.4%, *n* = 5/7), and calcification (85.7%, *n* = 6/7); (ii) the most common findings in CT were well-defined (77.8%, *n* = 7/9), heterogeneous (77.8%, *n* = 7/9), homo-hyperdense (66.7%, *n* = 6/9), and calcification (77.8%, *n* = 7/9); (iii) most patients (53.3%, *n* = 32/60) were symptomatic with a leading symptom of abdominal discomfort; (iv) most patients (56%, *n* = 28/50) received nonendoscopic treatment; (v) all patients (*n* = 60) did not recur after treatment; and (vi) CFT is statically different from GIST in age, size, and imaging calcification rate. Thus, CFT should be differentiated from GIST from clinical characteristics.

In our study, the ratio of children with CFT was 5%, which was statically different from GIST. One pooled population-based cohort study of GIST included 3480 patients with 0.37%, 14.9%, and 84.7% of ages 20, 20–50, and over 50 years, respectively [36]. However, the age distributions of 20, 20–50, and over 50 years were 5%, 40%, and 55% in our study, respectively, and were significantly different from GIST. We also compared the ratio of children and mean age between CFT and GIST, and both were significantly different (*p* < 0.001 and *p* < 0.037, respectively). The high ratio of children ratio with CFT may be because of genetic and/or embryologic factors. Fukunaga et al. discovered a diploid DNA in a 20-year-old female patient [37]. Trauma is a proposed predisposing factor of CFT, which might explain the high proportion of middle-aged patients [4].

Tumor size was significantly different between gastric CFT and GIST. This difference may be because gastric CFT has a benign character and grows slowly [31]. Most patients (53.3%) with CFT were symptomatic with the leading symptom of abdominal discomfort. Moreover, abdominal pain and abdominal distention were common. However, patients with GIST < 2 cm were asymptomatic, and those with GIST ≥ 2 cm were symptomatic with leading symptoms of GI bleeding and epigastric pain. Additionally, despite the small size (0.8 cm) of gastric CFT, they could be found easily.

Diagnostic methods include EUS, CT, fine-needle aspiration (FNA), and mucosal biopsy. Clinical diagnosis of CFT is challenging; however, some of these diagnostic methods may be helpful. EUS may help to differentiate CFT from GIST. CFT consists of hyalinized collagenization with scattered calcifications consistent with the most common findings in EUS (heterogeneous and iso-hypoechoic) rather than anechoic or hyperechoic in malignant GIST [38]. Moreover, more than half of gastric CFT is derived from the submucosa, and most GIST is derived from a muscle layer [38]. Frequently, the character of “calcifying” fibrous tumor will show high calcification rates in EUS, a rare finding in GIST [39]. A previous study showed that the rate of calcification in GIST is 3.6% (2/55) [40], significantly different from CFT.

The overall calcification rate of gastric CFT was 85.7% in this study, and all were macrocalcifications (on CT). However, macrocalcification in GIST is extremely rare; only a few case reports are published [41,42]. Therefore, calcification is the main character in the image finding of CFT. It is a reasonable way to differential diagnose between gastric CFT and GIST. EUS may have a higher sensitivity of calcification than CT on the image finding, and it could provide valuable information on macrocalcifications (coarse calcification) and preoperation.

The diagnostic yields of FNA in GIST are correlated with tumor size. The diagnosis of large gastric GIST in EUS-guided Trucut punch biopsies or EUS–FNA reached 50–70% [43]. In small GISTs (<2 cm), FNA, and treatment decisions in such circumstances may be a challenge [44]. EUS–FNA may be considered in GIST, especially for large ones, but it is not the standard management strategy. Two patients received FNA, but both failed to diagnose CFT [5,20] because it might have provided a small sample for diagnosis. Therefore, we do not suggest performing FNA in suspected CFT.

A frozen biopsy could not diagnose GIST; however, it could differentiate CFT from GIST. The cytomorphology of GIST is predominantly spindle cell (70%) or epithelioid (20%) [3]. In these 60 patients, only one patient with gastric CFT had a frozen biopsy and showed a benign fibrous lesion. However, frozen biopsies of nongastric CFTs might show inflammatory, fibrotic tissue [45], or hyalinized fibrosclerotic nodular lesions [46]. This distinctive cytomorphology may not be a firm diagnosis but could preliminarily differentiate the two diseases and avoid unnecessary surgery. However, only one case of gastric CFT had a frozen biopsy, but more data are needed. We concluded that a frozen biopsy in this situation could be valuable [12].

Finally, we suggested ESD for the treatment of gastric CFT. ESD appears to be a feasible and safe procedure in SMT [36]. In 2013, the first case of gastric CFT was treated with ESD [20]. Two patients were successfully treated with endoscopic full-thickness resection (EFR) in 2014 [22]. EFR may also be another choice for a patient with SMT arising from the muscle layer.

Occasionally, it is difficult to identify the exact resection area using conventional or laparoscopic surgery. Moreover, these surgeries may have operative complications, such as celialgia [32]. In this study, the patients who underwent a minor or major operative procedure showed a complication rate of 3.6% (*n* = 1/28), which was higher as compared to the endoscopic therapy (0%). In general, gastric CFT is a rare and benign tumor that can be easily removed by a simple excision procedure [4]. A total of 17 patients underwent endoscopic therapy, and no incidences of recurrence or complications were observed in any of the patients. All patients achieved adequate/expected treatment/outcome. These results highlight the suitability of ESD for treating gastric CFT.

Currently, limited knowledge is available for gastric CFT owing to the low number of reported cases (hundreds). There are a few limitations of this study that need to be addressed. Since no retrospective or prospective study was performed to compare the diagnosis between gastric CFT and GIST, the study and control groups may not have had adequate homogeneity. Moreover, only seven and nine patients were included in the EUS and CT image analysis, respectively, due to which selection bias could not be avoided. Additionally, since no study was performed to compare the choice of treatment in gastric CFT, the treatment suggestion was expert opinion. This study did not review non-English studies, and a total of 15 non-English articles (mostly in Japanese) were excluded. The retrospective nature of such studies makes them particularly susceptible to selection bias regarding the choice of patients that received interventions. In other words, patients that are diagnosed with gastric CFT but did not receive the intervention are not reported. Thus, making it difficult to determine the actual tumor size and demographics of gastric CFT.

Furthermore, no information is available regarding the exact rate of calcification for GIST. The calcification rate of GIST used in this study was approximately 3.6%, which was calculated on the basis of the information collected from relevant articles. Thus, there might be a discrepancy in the actual calcification rate of GIST and the data used for comparison with gastric CFT in this study.

In daily clinical practice, GIST is the most common SMT encountered by gastroenterologists and surgeons. The frequency of patients suffering from gastric CFT is comparatively less. Our detailed analysis suggests that a complete examination, including EUS and CT, should be performed for the cases of SMT that are suspected to be associated with GIST. This is particularly important for SMT cases involving children, middle-aged individuals (mean: 50 years old), small tumor sizes (~2 cm), third layer SMT observed in EUS, and calcification in EUS or CT. Further, these evaluations might provide additional information that will assist in distinguishing gastric CFT from GIST. In suspected cases of gastric CFT, a frozen biopsy performed during the endoscopic procedure might be helpful in differentiating a benign fibrous lesion from a spindle-cell lesion, except for the lesions that are large and the available sections might not be correct representatives. The management of such lesions depends on the final pathological diagnosis. The present study highlights the suitability of ESD/EFR in the diagnosis and differentiation of gastric CFT from GIST and treatment of gastric CFT regardless of the tumor size.

## 5. Conclusions

In conclusion, knowledge regarding certain patient characteristics, particularly age, tumor size, symptoms, imaging findings, and pathology, will be instrumental in distinguishing gastric CFT from GIST. Such a differential diagnosis prior to surgery will be helpful for gastroenterologists and surgeons in preventing excess surgery, especially in cases involving small tumor sizes, young age, submucosal lesion, and macrocalcification in CT images. Alternatively, a frozen biopsy might be helpful for diagnosis, except in cases where the lesions are large and the sections may not be representative. Such cases are managed depending on the final pathological diagnosis. We recommend the use of ESD for the differential diagnosis and treatment of gastric CFT, which will get low cost, less morbidity and mortality, non-invasive nature of ESD, less rate of complications, better quality of life observed in patients undergoing ESD.

## Figures and Tables

**Table 1:** Number of cases of providing available data.

Criteria	%	Number of Cases
Sex	100	60
Age	100	60
Symptom	100	60
Location	100	31
Layer	56.7	34
Size	98.3	59
Image		
EUS	11.7	7
CT	15	9
Choice of treatment	83.3	50
Follow-up	70	42
Recurrence	70	42

**Table 2:** Clinical characteristics of all patients.

Criteria	(Number of Cases)
**Sex** (M:F)	32:28 (60)
**Age** (year)	49.2 ± 13.9 (60)
Men	50.88 ± 14.04 (32)
Women	47.25 ± 13.85 (28)
Children ratio ^a^	5% (3)
**Symptom**	*n* = 60
Symptomatic	53.3% (32)
Acute	5% (3)
Chronic	16.7 % (10)
Asymptomatic	46.7% (28)
**Location**	*n* = 31
Antrum	6.6% (2)
Body	74.2% (23)
Fundus	19.4% (6)
**Layer**	*n* = 34
1st/2nd	14.7% (5)
3	55.9% (19)
4	23.5% (8)
5	5.9% (2)
**Size (cm)**	2.4 ± 3.15 (59)
**Image**	
EUS	*n* = 7
Heterogenous	100% (7)
Iso-hypoechoic	71.4% (5)
Hyperechoic	28.6% (2)
Well-defined	100% (7)
Acoustic shadowing (calcification)	85.7% (6)
CT	*n* = 9
Cannot detect tumor ^b^	21.4% (3)
Well-defined	77.8% (7)
Irregular	22.2% (2)
Homogeneous	22.2% (2)
Heterogeneous	77.8% (7)
Homo-hyperdense	66.7% (6)
Hypodense	33.3% (3)
Calcification	77.8% (7)
Macrocalcification (coarse)	85.7% (6)
Microcalcification (punctate)	14.3% (1)
**Overall calcification** ^c^	85.7% (12/14)
**Choice of treatment**	*n* = 50
Intervention	36% (18)
Diagnostic treatment	8% (4)
Minor operation (wedge resection)	44% (22)
Major operation	12% (6)
**Follow-up (Mon)**	24.1 ± 39.1 (42)
**Recurrence rate**	0% (0)

^a^: We defined children beyond 20 years. ^b^: 14 patients mentioned received computed tomography (CT), and two patients cannot find image, and three patients cannot detect the tumor. ^c^: Including endoscopic ultrasound (EUS) or CT. Two patients received both EUS and CT. One patient showed macrocalcification both in EUS and CT, and the other showed microcalcification in EUS but cannot show calcification in CT.

**Table 3:** Leading symptoms.

Symptoms	
Abdominal discomfort % (N)	55.6% (25)
Abdominal pain % (N)	17.8% (8)
Abdominal distention % (N)	8.9% (4)
Constitutional symptoms % (N)	4.4% (2)
Vomiting % (N)	4.4% (2)
GI bleeding % (N)	4.4% (2)
Retro-sternal burning sensation % (N)	4.4% (2)

**Table 4:** Distinctive preoperative surveys of gastric CFT and gastrointestinal stromal tumor (GIST).

Disease	Size (cm)	Age (yr)	Children (%)	Symptoms	Calcification (%)	Layer	EUS	CT
**CFT**	2.4	49.2	5	Mostly Sx (abdominal discomfort)	85.7	3rd	‧ Heterogeneous ‧ Iso-hypoechoic ‧ Calcification (85.7%)	‧ Well-defined ‧ Homo-hyperdense ‧ Calcification (77.8%)
**GIST**	6.0	65.0	0.5	<2 cm: usually Asx >2 cm: epigastric pain and GIB	3.6	4th	‧ Heterogeneous ‧ Hypoechoic ‧ Calcification (3.6%)	‧ <3 cm: ‧ Well-defined ‧ Homogeneous ‧ >3 cm: Lobulated ‧ Heterogeneous ‧ Irregular ‧ Calcification (3.6%)
***p* value**	<0.001	<0.001	0.037		<0.001		<0.001	<0.001
